# Evolving methods to combine cognitive and physical training for individuals with mild cognitive impairment: study protocol for a randomized controlled study

**DOI:** 10.1186/s13063-016-1650-4

**Published:** 2016-10-28

**Authors:** Ya-yun Lee, Ching-yi Wu, Ching-hung Teng, Wen-chuin Hsu, Ku-chou Chang, Poyu Chen

**Affiliations:** 1 School and Graduate Institute of Physical Therapy, College of Medicine, National Taiwan University, Taipei, Taiwan; 2 Department of Occupational Therapy and Graduate Institute of Behavioral Sciences, College of Medicine, Chang Gung University, 259 Wenhua 1st Rd, Taoyuan, 333 Taiwan; 3 Healthy Ageing Research Center, Chang Gung University, Taoyuan, Taiwan; 4 Department of Physical Medicine and Rehabilitation, Chang Gung Memorial Hospital at Linkou, Taoyuan, Taiwan; 5 Department of Neurology and Dementia Center, Chang Gung Memorial Hospital, Taoyuan, Taiwan; 6 Division of Cerebrovascular Diseases, Department of Neurology, Kaohsiung Chang Gung Memorial Hospital, Kaohsiung, Taiwan; 7 College of Medicine, Chang Gung University, Taoyuan, Taiwan

**Keywords:** Mild cognitive impairment, Physical exercise, Cognitive training, Hybrid therapy

## Abstract

**Background:**

Nonpharmacologic interventions, such as cognitive training or physical exercise, are effective in improving cognitive functions for older adults with mild cognitive impairment (MCI). Some researchers have proposed that combining physical exercise with cognitive training may augment the benefits of cognition. However, strong evidence is lacking regarding whether a combined therapy is superior to a single type of training for older adults with MCI. Moreover, which combination approach – combining physical exercise with cognitive training sequentially or simultaneously – is more advantageous for cognitive improvement is not yet clear. This proposed study is designed to clarify these questions.

**Methods/design:**

This study is a single-blinded, multicenter, randomized controlled trial. Eighty individuals with MCI will be recruited and randomly assigned to cognitive training (COG), physical exercise training (PE), sequential training (SEQ), and dual-task training (DUAL) groups. The intervention programs will be 90 min/day, 2–3 days/week, for a total of 36 training sessions. The participants in the SEQ group will first perform 45 min of physical exercise followed by 45 min of cognitive training, whereas those in the DUAL group will perform physical exercise and cognitive training simultaneously. Participants will be assessed at baseline, after the intervention, and at 6-month follow-up. The primary cognitive outcome tests will include the Montreal Cognitive Assessment and the color-naming Stroop test. Other outcomes will include assessments that evaluate the cognitive, physical, and daily functions of older adults with MCI.

**Discussion:**

The results of this proposed study will provide important information regarding the feasibility and intervention effects of combining physical exercise and cognitive training for older individuals with MCI.

**Trial registration:**

ClinicalTrials.gov Identifier: NCT02512627, registered on 20 July 2015.

## Background

Mild cognitive impairment (MCI) or minor neurocognitive disorder represents an intermediate stage between cognitive intactness and clinically diagnosed dementia [1, 2]. Although definite diagnostic criteria are lacking, individuals with MCI are characterized by self- or caregiver-reported memory and/or cognitive complaints, objective cognitive impairment, but independence in performing daily activities [1]. People with MCI can perform activities of daily living (ADLs) without assistance, but they appear to have a greater risk of falls and other adverse consequences [3].

Furthermore, MCI is a precursor of dementia or other severe cognitive disorders. The progression rate from MCI to dementia ranges from 10 to 15 % each year, and severe cognitive impairment or dementia will develop in more than 50 % of the MCI population within 5 years [1]. Therefore, providing early and appropriate interventions to individuals who manifest MCI may reduce the risk of adverse events, prevent cognitive decline, and ultimately, could decrease the burden of their caregivers and related medical expenses.

Clinical pharmacologic trials that used drugs to treat MCI have not been very successful [2, 4]. More encouraging results have been reported from nonpharmacologic treatments such as cognitive training or physical exercise [5, 6]. Cognitive training could enhance brain activation in the areas related to memory [5]. A systematic review has demonstrated that cognitive training programs have some positive effects on cognitive functions in older adults with and without MCI, especially on the specific tasks being practiced [7]. Physical exercise, on the other hand, had neuroprotective effects and lowered the risk of developing Alzheimer’s disease and related cognitive disorders [8]. A meta-analysis showed that in addition to health-related physical fitness and daily functions, physical exercise is favorable for unspecific cognitive abilities in older individuals with and without MCI [6]. It is thus suggested that physical exercise facilitates neuroplastic potential (e.g., neurogenesis) and that cognitive training induces positive neuroplastic changes for improving cognitive function [9].

Facilitation and guidance of neuroplasticity are two challenges of positive neuroplastic changes for cognitive gains. To overcome these two challenges, some recent studies have proposed that *combining physical exercise and cognitive training* may augment the intervention benefits on cognition for individuals with MCI [10, 11]. Animal studies have demonstrated that combining physical exercise and cognitive training promoted neurogenesis and angiogenesis, which resulted in greater cognitive improvement than exercise alone [12, 13]. Studies of community-dwelling older individuals without cognitive complaints found that combining physical exercise with cognitive training significantly improved memory and general cognitive functions compared with no intervention or a single type of training [10, 14].

Only a few studies have investigated the combined effects of physical exercise and cognitive training in people with MCI [15–18]. Barnes et al. recruited inactive older adults with cognitive complaints and found that the participants who received both aerobic exercise and mental activity training showed improvements in global cognitive function comparable to those who received a single mode (aerobic exercise only or mental activity only) of training [15]. Another study by Fiatarone Singh et al. [18] examined the sequential combination effects of progressive resistance training and cognitive training in older adults with MCI. Surprisingly and contrary to their initial hypotheses, the participants who received hybrid training improved *less* than those who participated in progressive resistance training alone or cognitive training alone [18].

Kounti et al. [16] and Suzuki et al. [17] designed specific therapeutic programs that combined physical and cognitive training in a dual-task format in which physical exercise and a cognitive task were performed simultaneously. Both studies found that the combined therapy induced significantly greater improvements on various cognitive functions in adults with MCI compared with the control groups that received health education programs [16, 17]. Another trial of dual-task training found that group-based training for 26 weeks improved global cognitive function in older adults with MCI compared with an exercise-only group, which highlighted the finding that group-based dual-task training enhanced global cognitive function [19].

As can be observed from this brief review, the treatment effects of combined physical exercise and cognitive training have varied across studies. The diverse treatment benefits could be attributed to different training approaches as well as to different combination methods – combining physical exercise and cognitive training sequentially or simultaneously – that are used in the studies. In addition, the control groups in those studies were not always dose-matched, did not involve active intervention, or were uneven (i.e., the study controlled for the exercise component but not the cognitive component). Hence, it is difficult to draw a consensus regarding (1) whether combining physical exercise and cognitive training is superior to a single mode of training for older individuals with MCI, and (2) which combination method (sequential versus simultaneous training) might result in better treatment outcomes.

Sequential training combines physical exercise and cognitive training sequentially. Studies conducted in rats/mice and in cognitively intact individuals have proposed that performing physical exercise before cognitive training may increase arousal level and thus facilitate cognitive task performance [20, 21]. Furthermore, physical exercise before cognitive training may enhance memory consolidation and influence the performance of memory retrieval [22, 23]. Winter et al. [24] examined the effects of a single bout of intense exercise on vocabulary learning and memory. The participants who engaged in intense exercise acquired the vocabularies 20 % faster than those who did not exercise or who engaged in low-intensity exercise. More interestingly, the intense exercise group also retained the vocabularies better than the control groups 1 week after the training [24]. Neurophysiologic measures revealed that exercise led to elevated levels of brain-derived neurotrophic factor and increased the cortical activation level of the prefrontal and occipital areas [20, 24], which are important for subsequent cognitive training.

In dual-task training, physical exercise and cognitive tasks are performed simultaneously. Dual tasking is one executive function that is especially important for ADLs. Recent studies revealed strong correlations between performance of ADLs and executive function in older adults [25]. Using functional near-infrared spectroscopy, Doi et al. found that increased prefrontal activation during dual tasking was correlated with executive functions in older adults with MCI [26]. Imaging studies in cognitively intact young and old adults also showed that behavioral improvement after dual-task training was associated with changes in prefrontal activation [27, 28]. The evidence from these studies suggests that an intervention using dual-task training could be a promising approach for individuals with MCI.

Behavioral and neurophysiologic evidence suggests that combining physical exercise and cognitive training sequentially or simultaneously has benefits on cognitive functions. However, strong evidence is lacking regarding which combination approach is more feasible, acceptable, and advantageous for older adults with MCI. This proposed randomized controlled trial is designed to address three specific aims: (1) to determine whether combined therapy, sequentially or simultaneously, is a feasible approach to train older adults with MCI, (2) to determine whether combined therapy can induce superior treatment outcomes compared with a single mode of intervention, and (3) to compare which combination approach – sequential or simultaneous – is more advantageous for improving cognitive functions, physical fitness, ADLs, and quality of life in adults with MCI.

## Methods/design

### Participants

We anticipate recruiting 80 participants from community facilities, day care centers, and nursing homes. The inclusion criteria are (1) men and women aged 60 years or older, (2) the ability to follow instructions, (3) objective cognitive impairment as measured by a Montreal Cognitive Assessment (MoCA) score < 26, (4) having self- or informant-reported memory or cognitive complaints, (5) the ability to perform ADLs, and (6) no diagnosed dementia [29]. Participants who have other neurologic disorders or an unstable medical condition (e.g., heart failure) that prevents them from performing physical exercise or cognitive training will be excluded. The participants will be randomly assigned to four groups: cognitive training (COG), physical exercise training (PE), sequential training (SEQ), and dual-task training (DUAL) groups. We specifically include the COG and PE groups as the “active control” groups to ensure that all participants can benefit from the study.

### Sample size estimation

No published study to date has compared the treatment effects of cognitive training, physical exercise training, sequential training, and dual-task training in older adults with MCI. Therefore, we estimated the sample size for the current study from the studies that compared sequential training or dual-task training with control intervention(s) in older adults with cognitive complaints [15–17]. Because our primary outcomes will include global cognitive function and executive function, we calculated the sample sizes on the outcomes that involve these cognitive aspects. Kounti et al. [16] found significant benefits of dual-task training on global cognitive function, as measured by the Mini-Mental State Examination (MMSE), in participants with MCI. Compared with the control intervention, the calculated effect size (Cohen’s *d*) was 0.63 for dual-task training [16]. Using G*Power software [30] with a power of 0.80 and a two-sided type I error of 0.05, the calculated total sample size for the current study will be 20 participants (i.e., 5 participants in each group). The study conducted by Suzuki et al. showed that compared with the control group, the multicomponent dual-task training group had greater improvement in MMSE after intervention [17]. Based on the calculated effect size for the MMSE (Cohen’s *d* = 0.45), we estimated the sample size for the current study will be 12 participants in each group. Barnes et al. investigated the treatment effects of a sequential combination of physical exercise and mental activity training and found significant benefits on the Useful Field of View (UFoV), a measure of executive function, in older adults with cognitive complaints [15]. We used the calculated effect size for the UFoV from Barnes’ study (Cohen’s *d* = 0.21) [15] to estimate a sample size of 32 participants in each group.

Similar to the study design conducted by Barnes et al. [15], we employ the active control groups (i.e., COG and PE groups) instead of a no-treatment or passive control group. On the other hand, the primary outcome measures used in the current study are closer to those used by Kounti et al. [16] and Suzuki et al. [17]. Hence, a mean value from the estimated sample sizes of the three studies was calculated, and we anticipated that recruiting 16 participants for each group should be sufficient for this proposed study.

The dropout rate in our pilot trial was approximately 20 % during the intervention period; hence, we plan to recruit 20 participants for each group resulting in a total sample size of 80 patients for four groups.

### Study design and procedures

**Fig. 1:**
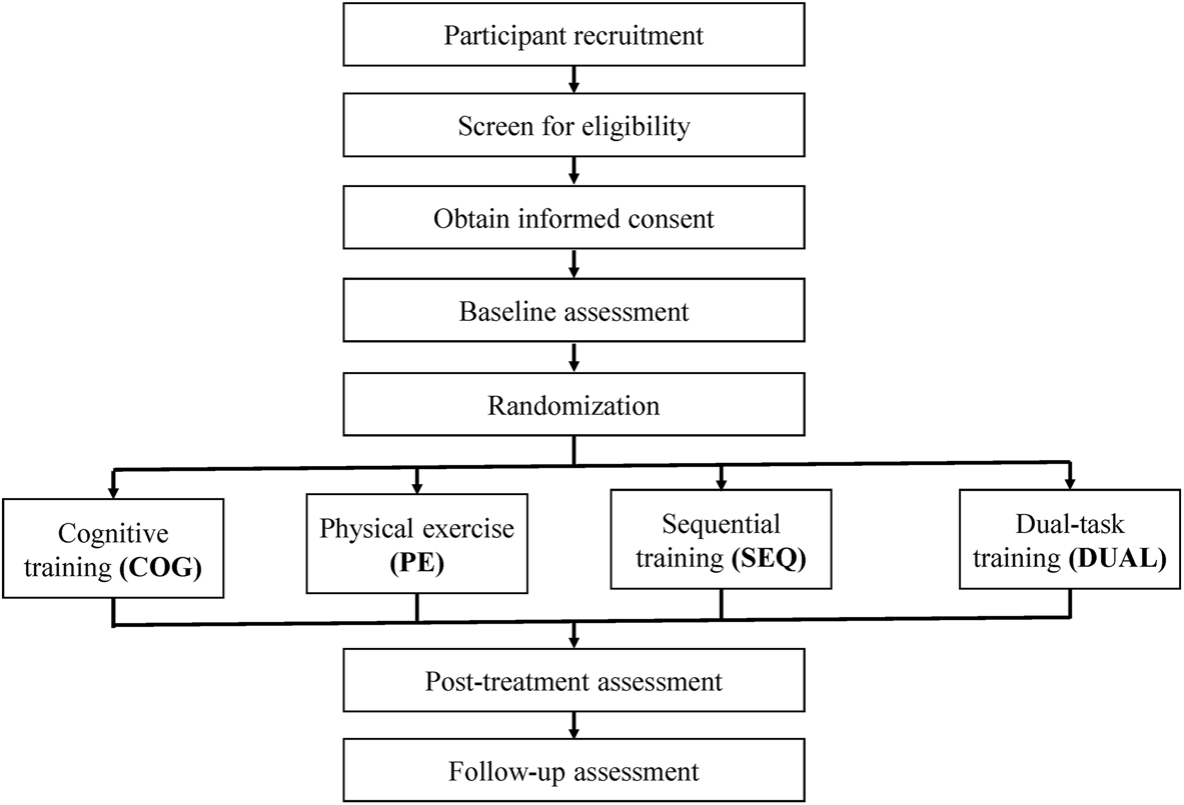
Flow diagram of the study

This proposed study is a randomized controlled trial with pretest, posttest, and 6-month follow-up assessments (Fig. 1). The study protocol is approved by the Chang Gung University Institutional Review Board. Intervention therapists will screen for potential participants and obtain signed informed consents from the eligible individuals before they participate in the study. The enrolled participants will be randomly assigned into the COG, PE, SEQ, or DUAL group with the allocation ratio of 1:1:1:1 [31]. Random tables based on the type of recruitment site (day care centers versus nursing homes versus community) will be generated by an independent research assistant using the web-based Research Randomizer tool (freely available at http://www.randomizer.org/) [32]. The research assistant will use the random table to decide the group allocation of a newly enrolled participant and will inform the relevant therapist to carry out the respective intervention. The intervention therapist will not know the group allocation until the pretest measurement is completed. Each intervention group will consist of three to five participants and will be guided by a trained therapist. The participants will engage in 90 min of training, two to three times a week, for a total of 36 training sessions.

Outcome measures, including cognitive functions, physical functions, ADLs, quality of life, and social participation, will be assessed before the treatment (pretest), after 36 training sessions (posttest), and 6 months after treatment (follow-up test). All outcome measures will be evaluated by independent evaluators who will be blinded to the group allocation of the participants. The evaluators will be trained by the principal investigator and senior therapists before the study begins to ensure that the measures will be conducted consistently. The assessments will be completed within 1 week before and after the intervention.

The first participant who adhered to the current study protocol was recruited in August 2015, although we piloted the study procedures in March 2015. We anticipate continuing to recruit until the sample size of 80 participants is reached.

### Interventions

The intervention therapists will be qualified occupational therapists or physical therapists, and will be trained by the principle investigator and the senior therapists to ensure standardized administration of the intervention protocols. The therapists will be required to complete a competency examination before providing the interventions. During the intervention period, the therapists will keep a daily log to record the progress and responses of each participant.

#### COG group

BrainHQ (Posit Science Inc., San Francisco, CA, USA) will be used to train different cognitive functions of the participants in the COG group. BrainHQ is a game-based computer program specifically designed to train various cognitive functions. This cerebral neuroplasticity-based computerized cognitive training program has been demonstrated to have significant benefits on various cognitive functions in community-dwelling older adults [33, 34]. During the training sessions, laptops with touchscreens will be provided to the participants so that they can play the computer-based games without having to manipulate a mouse. The BrainHQ games that involve cognitive functions of visuospatial processing, attention, memory, language, and logical thinking will be chosen for the participants to practice.

The participants will practice two to three games in 90 min, and the difficulty of the tasks will be adjusted to each individual's ability. The participants will not compete against each other but will play the games based on their own capability. The task will become more challenging as a participant improves. The intervention will contain 10 min of warm-up (explaining the rules), 70 min of cognitive training, and 10 min of cool-down (asking participants to recall and provide feedback to the activities). Each participant’s performance will be recorded in the daily log.

#### PE group

Previous studies suggested that a multimodal exercise program (aerobic with balance and strength training) may induce greater effects than a single mode of exercise [10, 35]. Therefore, we incorporate a multicomponent exercise program that includes balance training, strength training, and upper and lower limb aerobic exercise. The 90-min training session will be divided into two parts. In the first 45-min session, the participants will engage in aerobic exercise. The target intensity of the aerobic training will be 40 to 70 % of the maximal heart rate. Our pilot testing revealed that heart-related problems in some older people may prohibit them from achieving an aerobic level of exercise if measured by maximal heart rate. Thus, we will also adopt a Borg rating of perceived exertion (RPE) score of 12 to 14 as an indicator of achieving the aerobic level [36]. The therapists will adjust the aerobic level according to the capability of each participant, and the participants can rest as needed.

In the second 45-min session, the participants will engage in balance training and muscle strengthening. Static and dynamic balance training will be carried out under sitting and standing positions. Thera-Bands and weights will be used for the muscle-strengthening programs of the upper and lower limb muscles. The order of balance training and muscle strengthening will be intermixed. Each 45-min training session will contain 5 min of warm-up, 35 min of physical exercise, and 5 min of cool-down. The exercises will be performed without ergometers.

#### SEQ group

The participants in the SEQ group will first perform 45 min of physical exercise, followed by 45 min of cognitive training. During the physical exercise session, the participants will engage in 5 min of warm-up, 35 min of physical exercise, and 5 min of cool-down. The physical exercise programs will involve 15 to 17 min of aerobic exercise and 18 to 20 min of balance training and muscle strengthening. During the cognitive training session, the intervention therapists will spend 5 min explaining the rules of the tasks. Then, the participants will engage in 35 min of cognitive training with the BrainHQ games, as described in the COG group. The participants will be given one or two games to practice during the training session. The session will end with 5 min of cool-down activities such as proving feedback and asking the participants to recall the activities.

#### DUAL group

**Fig. 2:**
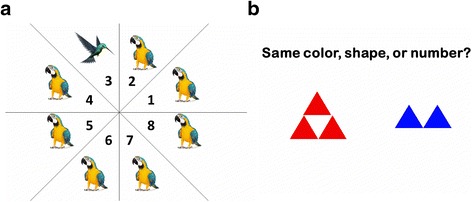
Examples of the PowerPoint versions of BrainHQ games. **a** Hawk eye: search for a different bird and determine the location of that bird. **b** Divided attention: determine whether the two figures have the same color, shape, or number

Participants in the DUAL group will be instructed to perform physical exercise and cognitive tasks simultaneously. Because dual-task training is a more challenging approach for participants with MCI, we have used PowerPoint presentations to design simpler versions of the BrainHQ games to make sure that the participants will be able to follow the instructions and engage in the games (Fig. 2). Similar to the COG and SEQ groups, the difficulty of the cognitive tasks will be adjusted as the participants improve in their performance. The physical exercises involved in this group will be carefully chosen to ensure that our participants can effectively perform the exercises while focusing on the cognitive tasks. The exercise programs will also include the components of aerobic exercise (e.g., stepping in place), balance training (e.g., static standing or walking), and muscle strengthening (e.g., using Thera-Band or weights). Examples of the dual-task training programs include practicing math calculations while stepping and search for a different bird on the screen (Fig. 2a) while strengthening the hip muscles with a Thera-Band.

During the 90-min dual-task training session, the participants will engage in two combinations of physical activity and cognitive training program. For each combination, the intervention therapists will spend 10 min explaining the cognitive task and the method to perform the physical activity, followed by 35 min of training.

### Outcome measures

Sociodemographic variables, including age, gender, education level, comorbidity, smoking, alcohol intake, and depressive symptoms, will be recorded during the initial evaluation. This study will assess the participants before, immediately after, and 6 months after the intervention programs. The outcome measures will include several domains: cognitive functions, physical functions, ADLs, quality of life, and social participation.

#### Primary outcome measures

The MoCA will be used to assess general cognitive function. The MoCA examines visuospatial processing, naming, verbal fluency, abstract concept, short-term memory, digit forward and backward span, counting, and orientation. The total score for the MoCA is 30, with a higher number indicating a more intact cognitive function. The MoCA has been shown to be a valid and promising tool to detect MCI and early Alzheimer’s disease [37].

The color-naming Stroop test will be used to assess the abilities of inhibition, set-shifting, and selective attention [19]. The participants will be tested under congruent and incongruent conditions. In the congruent condition, the ink color of a word is consistent with the written color name; however, the ink color differs from the written color name under the incongruent condition. The participants will be required to read out the ink color of the word (but not the word itself) as accurately and as soon as possible. The time to complete the task will be calculated for each condition [38, 39].

#### Secondary outcome measures

##### Cognitive functions

Subtests of the Wechsler Adult Intelligence Scale (WAIS) and the Wechsler Memory Scale (WMS) will be used to measure memory and cognitive functions. The subtests of WAIS will include digit symbol-coding (total score = 133) and matrix reasoning (total scale = 26). The WMS subtests will consist of facial recognition (total scale = 48), verbal paired associates (total scale = 32), word lists (total scale = 48), and spatial span (total scale = 32). The WAIS and the WMS have high reliability and validity and are often used to differentiate individuals with memory or cognitive deficits from those who are cognitively intact. The WAIS and the WMS can also be used to assess cognitive improvements after a specific intervention [40].

Useful Field of View (UFoV) is the visual area over which useful information can be obtained at a quick glance without eye or head movements. This UFoV will be assessed with the *Double Decision* game in the BrainHQ program. The participants will be asked to select a particular symbol shape located at the center while remembering the position of another image presented on the screen. The UFoV requires the abilities of visuomotor processing, divided attention, and selective attention [41].

Dual-task tests will be used to assess an individual’s ability to perform two tasks simultaneously. The dual-task test could reflect the attentional limitation, central executive ability, and automatic processing ability of the participant [42, 43]. The dual-task performance will be evaluated while the individual performs the 10-m Walk Test and the Box and Block Test (BBT). For the 10-m Walk Test, the participants will be asked to walk 10 m at their comfortable walking speed. The walking time and number of steps will be recorded. The BBT will be assessed with a wooden box containing two equally sized compartments. Cubes will be placed in one compartment, and the participants will be instructed to use their dominant hand to move the cubes to the other compartment one-by-one at their fastest speed. The number of cubes moved within 60 s will be recorded. The secondary cognitive task that will be performed during the 10-m Walk Test or BBT will be the counting task and the response time task. For the math calculation, the participants will be required to count backward by 7 from a given number (e.g., 100 or 200); for the response time task, the participants will be required to listen to a series of sounds and respond to a high- or low-pitch tone as fast as possible. The results of the dual-task tests will provide information about whether the two tasks compete for the same class of neural resources or one of the tasks can be carried out automatically [44].

##### Physical functions

The Timed Up and Go (TUG) test will be used to assess mobility and dynamic balance ability. The participants will be required to stand up from a chair, walk 3 m at their comfortable speed, turn around, then walk back to the chair and sit down. The time to complete the TUG test has been shown to be a good indicator to detect potential people who are likely to fall and frail older people [45]. The test-retest reliability of the TUG on individuals with cognitive impairment is excellent [46].

The 30-s Chair-Stand Test (CST) will be assessed to indicate the strength and endurance of the lower extremities. The participants are asked to stand up from a standardized chair and then sit down as many times as possible within 30 s. The feasibility and reliability of using the CST in people with cognitive impairment have been established to be good [46].

The Chinese version of the International Physical Activity Questionnaires (IPAQ) is an international measure for health-related physical activity. This study will use a short form of the IPAQ to assess changes in physical activity before and after the intervention. The reliability and validity of the IPAQ has been established across 12 countries [47].

ActiGraph GX3 accelerometers (ActiGraph, Shalimar, FL, USA) will be used to measure physical activity outside the laboratory settings [48, 49]. The participants will be asked to wear the actigraphy on both wrists all day for three consecutive weekdays before and after the intervention. The number and average counts of movement per minute for the participants will be recorded and calculated by the accelerometer. Data recorded by the actigraphy will be analyzed using MAHUFFE software (http://www.mrc-epid.cam.ac.uk/). The use of actigraphy to measure arm use and physical activity has been established for older people [50].

Decreased muscle mass, muscle strength, and movement quality are related to the aging process that will affect physical activities in older adults [51]. Therefore, muscle strength will also be recorded on both sides for knee extension, knee flexion, and grip strength using a handheld dynamometer (Commander Power Track Muscle Dynamometer Manual Muscle Tester, JTECH Medical) and a Jamar Handgrip Dynamometer.

##### ADLs, quality of life, and social participation

The ADLs will be assessed with the Chinese version of the Disability Assessment for Dementia (DAD), a questionnaire for evaluating the basic ADLs and instrumental ADLs in participants in the early stages of dementia. The DAD subtests include hygiene, dressing, continence, eating, meal preparation, telephoning, going on an outing, finance and correspondence, medications, and leisure and housework. The DAD assesses an individual’s performance from the perspectives of initiation, planning and organization, and effective performance [31].

In addition, the Chinese versions of the Barthel Index (BI) and the Lawton Instrumental Activities of Daily Living Scale (Lawton IADL) will be assessed. These questionnaires measure the performance of feeding, grooming, bathing, toileting, transferring, and tool using, among others. The BI and the Lawton IADL are both commonly used to evaluate older adults with cognitive impairment [52].

The treatment effects on quality of life will be assessed with the Chinese versions of the Quality of Life in Alzheimer’s Disease Instrument (QoLAD), the Caregiver Burden Inventory (CBI), and the short form of the Geriatric Depression Scale (GDS). The Chinese versions of these assessments have good reliability and validity [53–55].

Finally, social participation will be assessed with the Community Integration Questionnaire (CIQ), which measures items relevant to home integration, social integration, and productive activities [56]. The reliability and validity of the CIQ have been established in patients with traumatic brain injuries and stroke [56, 57].

### Data analysis

We will use analysis of variance (ANOVA) and χ^2^ to analyze differences in baseline characteristics and baseline outcome measures among the groups. Any differences among the groups in the baseline sociodemographic variables will become covariates in the subsequent statistical analysis. Repeated measures ANOVA will be used to determine the intervention effects for the four groups. A significant statistical level will be set at 0.05 for all comparisons. Besides *p* values, effect size η^2^ will also be calculated to determine the group difference for each outcome measure. An η^2^ greater than 0.138 is considered to be a large effect, an η^2^ between 0.138 and 0.059 is a moderate effect, and an η^2^ between 0.01 and 0.059 is a small effect [58]. Intention-to-treat will be used for missing data. Data analysis will be performed with PASW Statistics 18.0 software (IBM Corp., Armonk, NY, USA).

### Data management and monitoring

Outcome measures will be recorded with pen-and-paper during assessment. A trained research assistant will manually enter the data into an electronic database. A second research assistant independent from this study will check the quality and accuracy. The paper data collection sheets and signed informed consents will be stored in a locked cabinet, and the electronic database will be password-protected. Any unanticipated adverse events that occur during the course of data collection will be reported to the Institutional Review Board in accordance with the procedures of Chang Gung University, Taoyuan, Taiwan. For any revised study procedure, the modification will be submitted to the Chang Gung University Institutional Review Board for approval and to ClinicalTrials.gov. The data will be kept confidential with only limited access to research investigators and related research assistants and graduate students.

### Potential adverse events

One potential adverse event that may occur during the intervention is an increased risk of falls when physical exercises are performed in the standing position. To minimize the risks of falls, the intervention therapists will ensure that group exercises are performed while sitting. When the participants are required to stand up or walk around to enhance their balance abilities, one-on-one therapy will be conducted to ensure the participants’ safety.

## Discussion

The goals of this proposed study are to first determine whether the combined therapy – joint physical exercise and cognitive training – is a feasible approach for training older people with MCI and, second, to investigate whether the combined therapy will result in superior cognitive, physical, ADLs, and quality of life outcomes compared with exercise or cognitive training alone. Some studies have used combined therapy to train adults with MCI, but the results were inconsistent. Although some studies found that the combined therapy is more advantageous for cognitive functions than a single mode of training, some reported no superior benefits [10]. Fiatarone Singh et al. (2014) even observed that the participants who received combined intervention showed less improvement in cognitive functions than those who had received exercise training alone or cognitive training alone. This result generated the hypothesis that the combined approach may cause excessive mental and physical stress to the participants, leading to less favorable outcomes [18]. Because most adults with MCI are at an older age, with possibly several chronic disorders (e.g., hypertension, diabetes, etc.), probing the feasibility and acceptability of the combined therapy is necessary and important.

This proposed project aims to move the field forward by comparing the intervention effects of different combination approaches – combining physical exercise and cognitive training sequentially or simultaneously – with a single type of training. Both combination approaches have been investigated, but which hybrid method could result in superior outcomes is not yet clear. Some have suggested that performing physical exercise before cognitive training could increase the arousal level for subsequent cognitive tasks [20, 21] and enhance memory consolidation and retrieval [22, 23]. These cognitive benefits were associated with elevated levels of brain-derived neurotrophic factor [24] and increased cortical activation level of the prefrontal and occipital areas [20]. Dual-task training also activated the prefrontal areas in older adults with MCI [26], and the changes in prefrontal activation were associated with improvements in executive functions [27, 28]. It has been postulated that cerebellar activation would be stronger when individuals perform two tasks simultaneously and could, therefore, lead to greater training benefits for the learned task [59].

This study is distinctive for comparing the treatment effects of various dose-matched interventions in older adults with MCI. Schaefer and Schumacher emphasized the importance of equal treatment time when designing a randomized controlled trial for older individuals with MCI [59]. This study has, therefore, designed a comprehensive assessment and intervention protocol to investigate the treatment effects of different combined therapies in people with MCI.

One big challenge that we may encounter for the study is the difficulty in recruiting participants and another is the ability to maintain those participants. Recruiting older adults who have some cognitive impairment, but do not have other neurologic or psychological disorders, could be very challenging. Those older individuals typically stay in their home community and maintain a fairly active life before they become too old to carry out household tasks. To address this problem, we will target older adults who are sent to community day care centers and some nursing homes that admit older individuals without caregivers. Furthermore, the older adults who agree to participate in the study may decide to discontinue from the program during the 36 training sessions. Dropping out of the intervention program may be due to personal reasons (e.g., falls, diseases, or low motivation) or family issues (e.g., moving, need to take care of their partner, or change in caregivers) that may prohibit them from continuing the study. We accounted for the dropout rate in the sample size estimation to ensure that we could reach sufficient power for the study.

The overall goal of this study is to compare the treatment effects of different combinations of physical exercise and cognitive training. The results of this study will be important for clinicians as well as family caregivers to select the most efficient and effective training approach to improve cognitive, physical, and daily functions in older individuals with MCI. Most importantly, the ultimate goal of these interventions is to enhance their ability to live independently and to improve their quality of life.

## Trial status

The trial has currently recruited 39 patients since August 2015, and 12 participants have completed the 6-month follow-up test. We will continue to recruit until the sample size of 80 participants is reached.

## Abbreviations

ADLs: Activities of daily living
BBT: Box and Block Test
CBI: Caregiver Burden Inventory
CIQ: Community Integration Questionnaire
CST: Chair-stand Test
DAD: Disability Assessment for Dementia
GDS: Geriatric Depression Scale
IPAQ: International Physical Activity Questionnaires
Lawton IADL: Lawton Instrumental Activities of Daily Living Scale
MCI: Mild cognitive impairment
QoLAD: the Quality of Life in Alzheimer’s Disease Instrument
TUG: Timed Up and Go
UFoV: Useful Field of View
WAIS: Wechsler Adult Intelligence Scale
WMS: Wechsler Memory Scale

## References

[1] Petersen RC, Caracciolo B, Brayne C, Gauthier S, Jelic V, Fratiglioni L (2014). Mild cognitive impairment: a concept in evolution. J Intern Med.

[2] Gauthier S, Reisberg B, Zaudig M, Petersen RC, Ritchie K, Broich K (2006). Mild cognitive impairment. Lancet.

[3] Montero-Odasso M, Wells JL, Borrie MJ, Speechley M (2009). Can cognitive enhancers reduce the risk of falls in older people with mild cognitive impairment? A protocol for a randomised controlled double blind trial. BMC Neurol.

[4] Petersen RC, Thomas RG, Grundman M, Bennett D, Doody R, Ferris S (2005). Vitamin E and donepezil for the treatment of mild cognitive impairment. N Engl J Med.

[5] Simon SS, Yokomizo JE, Bottino CM (2012). Cognitive intervention in amnestic mild cognitive impairment: a systematic review. Neurosci Biobehav Rev.

[6] Heyn P, Abreu BC, Ottenbacher KJ (2004). The effects of exercise training on elderly persons with cognitive impairment and dementia: a meta-analysis. Arch Phys Med Rehabil.

[7] Jean L, Bergeron ME, Thivierge S, Simard M (2010). Cognitive intervention programs for individuals with mild cognitive impairment: systematic review of the literature. Am J Geriatr Psychiatry.

[8] Laurin D, Verreault R, Lindsay J, MacPherson K, Rockwood K (2001). Physical activity and risk of cognitive impairment and dementia in elderly persons. Arch Neurol.

[9] Bamidis PD, Vivas AB, Styliadis C, Frantzidis C, Klados M, Schlee W (2014). A review of physical and cognitive interventions in aging. Neurosci Biobehav Rev.

[10] Law LL, Barnett F, Yau MK, Gray MA (2014). Effects of combined cognitive and exercise interventions on cognition in older adults with and without cognitive impairment: a systematic review. Ageing Res Rev.

[11] Gates NJ, Valenzuela M, Sachdev PS, Singh NA, Baune BT, Brodaty H (2011). Study of Mental Activity and Regular Training (SMART) in at risk individuals: a randomised double blind, sham controlled, longitudinal trial. BMC Geriatr.

[12] Fabel K, Wolf SA, Ehninger D, Babu H, Leal-Galicia P, Kempermann G (2009). Additive effects of physical exercise and environmental enrichment on adult hippocampal neurogenesis in mice. Front Neurosci.

[13] Langdon KD, Corbett D (2012). Improved working memory following novel combinations of physical and cognitive activity. Neurorehabil Neural Repair.

[14] Fabre C, Chamari K, Mucci P, Masse-Biron J, Prefaut C (2002). Improvement of cognitive function by mental and/or individualized aerobic training in healthy elderly subjects. Int J Sports Med.

[15] Barnes DE, Santos-Modesitt W, Poelke G, Kramer AF, Castro C, Middleton LE (2013). The Mental Activity and eXercise (MAX) trial: a randomized controlled trial to enhance cognitive function in older adults. JAMA Intern Med.

[16] Kounti F, Bakoglidou E, Agogiatou C, Lombardo NBE, Serper LL, Tsolaki M (2011). RHEA, a nonpharmacological cognitive training intervention in patients with mild cognitive impairment: a pilot study. Top Geriatr Rehabil.

[17] Suzuki T, Shimada H, Makizako H, Doi T, Yoshida D, Tsutsumimoto K (2012). Effects of multicomponent exercise on cognitive function in older adults with amnestic mild cognitive impairment: a randomized controlled trial. BMC Neurol.

[18] Fiatarone Singh MA, Gates N, Saigal N, Wilson GC, Meiklejohn J, Brodaty H (2014). The Study of Mental and Resistance Training (SMART) study-resistance training and/or cognitive training in mild cognitive impairment: a randomized, double-blind, double-sham controlled trial. J Am Med Dir Assoc.

[19] Gill DP, Gregory MA, Zou G, Liu-Ambrose T, Shigematsu R, Hachinski V (2016). The healthy mind, healthy mobility trial: a novel exercise program for older adults. Med Sci Sports Exerc.

[20] Li L, Men WW, Chang YK, Fan MX, Ji L, Wei GX (2014). Acute aerobic exercise increases cortical activity during working memory: a functional MRI study in female college students. PLoS One.

[21] Roig M, Skriver K, Lundbye-Jensen J, Kiens B, Nielsen JB (2012). A single bout of exercise improves motor memory. PLoS One.

[22] Coles K, Tomporowski PD (2008). Effects of acute exercise on executive processing, short-term and long-term memory. J Sports Sci.

[23] Labban JD, Etnier JL (2011). Effects of acute exercise on long-term memory. Res Q Exerc Sport.

[24] Winter B, Breitenstein C, Mooren FC, Voelker K, Fobker M, Lechtermann A (2007). High impact running improves learning. Neurobiol Learn Mem.

[25] Eggenberger P, Theill N, Holenstein S, Schumacher V, de Bruin ED (2015). Multicomponent physical exercise with simultaneous cognitive training to enhance dual-task walking of older adults: a secondary analysis of a 6-month randomized controlled trial with 1-year follow-up. Clin Interv Aging.

[26] Doi T, Makizako H, Shimada H, Park H, Tsutsumimoto K, Uemura K (2013). Brain activation during dual-task walking and executive function among older adults with mild cognitive impairment: a fNIRS study. Aging Clin Exp Res.

[27] Erickson KI, Colcombe SJ, Wadhwa R, Bherer L, Peterson MS, Scalf PE (2007). Training-induced functional activation changes in dual-task processing: an FMRI study. Cereb Cortex.

[28] Erickson KI, Colcombe SJ, Wadhwa R, Bherer L, Peterson MS, Scalf PE (2007). Training-induced plasticity in older adults: effects of training on hemispheric asymmetry. Neurobiol Aging.

[29] Petersen RC (2004). Mild cognitive impairment as a diagnostic entity. J Intern Med.

[30] Faul F, Erdfelder E, Lang AG, Buchner A (2007). G*Power 3: a flexible statistical power analysis program for the social, behavioral, and biomedical sciences. Behav Res Methods.

[31] Hooghiemstra AM, Eggermont LH, Scheltens P, van der Flier WM, Bakker J, de Greef MH (2012). Study protocol: EXERcise and cognition in sedentary adults with early-ONset dementia (EXERCISE-ON). BMC Neurol.

[32] Urbaniak GC, Plous S. Research Randomizer (Version 4.0) [Computer software]. 2013. http://www.randomizer.org/. Accessed 22 June 2013.

[33] Smith GE, Housen P, Yaffe K, Ruff R, Kennison RF, Mahncke HW (2009). A cognitive training program based on principles of brain plasticity: results from the Improvement in Memory with Plasticity-based Adaptive Cognitive Training (IMPACT) study. J Am Geriatr Soc.

[34] Zelinski EM, Spina LM, Yaffe K, Ruff R, Kennison RF, Mahncke HW (2011). Improvement in memory with plasticity-based adaptive cognitive training: results of the 3-month follow-up. J Am Geriatr Soc.

[35] Erickson KI, Kramer AF (2009). Aerobic exercise effects on cognitive and neural plasticity in older adults. Br J Sports Med.

[36] Borg G, Hassmen P, Lagerstrom M (1987). Perceived exertion related to heart rate and blood lactate during arm and leg exercise. Eur J Appl Physiol Occup Physiol.

[37] Nasreddine ZS, Phillips NA, Bedirian V, Charbonneau S, Whitehead V, Collin I (2005). The Montreal Cognitive Assessment, MoCA: a brief screening tool for mild cognitive impairment. J Am Geriatr Soc.

[38] Koss E, Ober BA, Delis DC, Friedland RP (1984). The Stroop color-word test: indicator of dementia severity. Int J Neurosci.

[39] Ridley DR, Johnson DE, Braisted PD (1978). The color-word connotative incongruity effect. Percept Mot Skills.

[40] Iverson GL (2001). Interpreting change on the WAIS-III/WMS-III in clinical samples. Arch Clin Neuropsychol.

[41] Ball K, Edwards JD, Ross LA (2007). The impact of speed of processing training on cognitive and everyday functions. J Gerontol B Psychol Sci Soc Sci.

[42] Pashler H (1994). Dual-task interference in simple tasks: data and theory. Psychol Bull.

[43] Plummer-D’Amato P, Altmann LJ, Saracino D, Fox E, Behrman AL, Marsiske M (2008). Interactions between cognitive tasks and gait after stroke: a dual task study. Gait Posture.

[44] Remy F, Wenderoth N, Lipkens K, Swinnen SP (2010). Dual-task interference during initial learning of a new motor task results from competition for the same brain areas. Neuropsychologia.

[45] Podsiadlo D, Richardson S (1991). The timed “Up & Go”: a test of basic functional mobility for frail elderly persons. J Am Geriatr Soc.

[46] Blankevoort CG, van Heuvelen MJ, Scherder EJ (2013). Reliability of six physical performance tests in older people with dementia. Phys Ther.

[47] Craig CL, Marshall AL, Sjostrom M, Bauman AE, Booth ML, Ainsworth BE (2003). International physical activity questionnaire: 12-country reliability and validity. Med Sci Sports Exerc.

[48] Domene PA, Easton C (2014). Combined triaxial accelerometry and heart rate telemetry for the physiological characterization of Latin dance in non-professional adults. J Dance Med Sci.

[49] Strath SJ, Greenwald MJ, Isaacs R, Hart TL, Lenz EK, Dondzila CJ (2012). Measured and perceived environmental characteristics are related to accelerometer defined physical activity in older adults. Int J Behav Nutr Phys Act.

[50] Johnson LG, Butson ML, Polman RC, Raj IS, Borkoles E, Scott D (2016). Light physical activity is positively associated with cognitive performance in older community dwelling adults. J Sci Med Sport.

[51] Rantanen T, Avlund K, Suominen H, Schroll M, Frandin K, Pertti E (2002). Muscle strength as a predictor of onset of ADL dependence in people aged 75 years. Aging Clin Exp Res.

[52] van der Putten JJ, Hobart JC, Freeman JA, Thompson AJ (1999). Measuring change in disability after inpatient rehabilitation: comparison of the responsiveness of the Barthel index and the Functional Independence Measure. J Neurol Neurosurg Psychiatry.

[53] Yu HM, He RL, Ai YM, Liang RF, Zhou LY (2013). Reliability and validity of the Quality of Life-Alzheimer Disease Chinese version. J Geriatr Psychiatry Neurol.

[54] Chou KR, Jiann-Chyun L, Chu H (2002). The reliability and validity of the Chinese version of the Caregiver Burden Inventory. Nurs Res.

[55] Wong MT, Ho TP, Ho MY, Yu CS, Wong YH, Lee SY (2002). Development and inter-rater reliability of a standardized verbal instruction manual for the Chinese Geriatric Depression Scale-short form. Int J Geriatr Psychiatry.

[56] Willer B, Ottenbacher KJ, Coad ML (1994). The Community Integration Questionnaire. A comparative examination. Am J Phys Med Rehabil.

[57] Dalemans RJ, de Witte LP, Beurskens AJ, van den Heuvel WJ, Wade DT (2010). Psychometric properties of the Community Integration Questionnaire adjusted for people with aphasia. Arch Phys Med Rehabil.

[58] Portney LG, Watkins MP (2009). Power and sample size. Foundations of clinical research: applications to practice.

[59] Schaefer S, Schumacher V (2011). The interplay between cognitive and motor functioning in healthy older adults: findings from dual-task studies and suggestions for intervention. Gerontology.

